# Self-Organization Dynamics of Collagen-like Peptides Crosslinking Is Driven by Rose-Bengal-Mediated Electrostatic Bridges

**DOI:** 10.3390/pharmaceutics14061148

**Published:** 2022-05-27

**Authors:** Roberto Rosales-Rojas, Matías Zuñiga-Bustos, Francisca Salas-Sepúlveda, Constanza Galaz-Araya, Ricardo A. Zamora, Horacio Poblete

**Affiliations:** 1 Center for Bioinformatics, Simulation and Modelling, Facultad de Ingeniería, Universidad de Talca, Talca 3465548, Chile; roberto.rosales@utalca.cl (R.R.-R.); matiaszunigabustos@gmail.com (M.Z.-B.); fsalas17@alumnos.utalca.cl (F.S.-S.); constanza.galaz@utalca.cl (C.G.-A.); 2 Doctorado en Ciencias mención Modelado de Sistemas Químicos y Biológicos, Facultad de Ingeniería, Universidad de Talca, Talca 3465548, Chile; 3 Institute for Bioengineering of Catalonia (IBEC), The Barcelona Institute of Science and Technology, Baldiri Reixac 10-12, 08028 Barcelona, Spain; 4 Network Biomedical Research Center on Bioengineering, Biomaterials and Nanomedicine (CIBER-BBN), 28029 Madrid, Spain

**Keywords:** collagen-like peptide, Rose Bengal, crosslinking, photochemical tissue bonding therapies, molecular dynamics, QM/MM simulations

## Abstract

The present work focuses on the computational study of the structural micro-organization of hydrogels based on collagen-like peptides (CLPs) in complex with Rose Bengal (RB). In previous studies, these hydrogels computationally and experimentally demonstrated that when RB was activated by green light, it could generate forms of stable crosslinked structures capable of regenerating biological tissues such as the skin and cornea. Here, we focus on the structural and atomic interactions of two collagen-like peptides (collagen-like peptide I (CLPI), and collagen-like peptide II, (CLPII)) in the presence and absence of RB, highlighting the acquired three-dimensional organization and going deep into the stabilization effect caused by the dye. Our results suggest that the dye could generate a ternary ground-state complex between collagen-like peptide fibers, specifically with positively charged amino acids (Lys in CLPI and Arg in CLPII), thus stabilizing ordered three-dimensional structures. The discoveries generated in this study provide the structural and atomic bases for the subsequent rational development of new synthetic peptides with improved characteristics for applications in the regeneration of biological tissues during photochemical tissue bonding therapies.

## 1. Introduction

Chemical, physical, and enzymatic crosslinking methods have been widely used to increase the mechanical properties of collagen triple helices and collagen-like peptides triple helices (CLP) in tissue regeneration therapies. For years, non-active light bonding molecules for the intra- and inter-chemical crosslinking of CLPs were successfully used to generate tissue-engineered collagen scaffolds with physicochemical and mechanical properties such as those found in cellular tissues. Tris (2-aminoethyl) amine/succinic acid [1], oxime [2], glutaraldehyde, formaldehyde, 1-ethyl-3-(3-dimethylaminopropyl) carbodiimide hydrochloride/N-hydroxysuccinimide [3], and CLP modified with terminal cysteines [4] are some of the most used non-active light-bonding molecules to date.

Currently, photochemical tissue bonding (PTB) therapy is one of the most promising techniques in wound repair treatment. The basis of the PTB is the use of active light bonding molecules (photosensitizer, PS) that can promote the covalent crosslink of proteins by generating radicals with specific lighting. Using a PS, PTB has been successfully used in in vitro, ex vivo and in vivo models [5,6,7,8,9].

Compared to suturing method, the use of PTB generates minimal scarring and fibrosis, and produces less inflammatory effects [10,11,12]. Porphyrins, phthalocyanines, phenothiazines, and xanthenes have been widely used to generate collagen crosslinking in tissues, such as articular cartilage, skin, and cornea [13,14]. In this sense, Rose Bengal (RB), belonging to the Xanthene family, is a noteworthy PS dye used as a light-activated tissue-bonding molecule due to its recent use in cornea repair therapy for human patients [15]. 

Initially, RB was used as PS in photodynamic therapies (PDT) for cancer treatment [16]. RB was also used in cancer treatment due to its property of being able to be activated by ultrasound, causing ultrasonic cavitation, which can induce lysis, necrosis, or apoptosis [16].

Interestingly, in PDT, RB presented a higher phototoxicity in skin-cultured cells compared to cells in the skin or dermal fibroblasts cultured in a collagen gel matrix [17]. On the other hand, RB presents phototoxicity in corneal keratocytes compared to dermal fibroblast [17]. The main difference in phototoxicity was suggested to be the presence of collagen. 

The binding of RB to collagen fibers decreased their bioavailability for entry into cells [17]. It was previously reported that the RB photodegradation rate was lower in collagen-containing solutions; it allowed the material to establish interactions with healthy tissue and promote tissue regeneration [18]. 

Currently, the basic proposed mechanism is the ability of RB to interact electrostatically with collagen fibers and promote the generation of radicals in these fibers, which can subsequently interact and generate covalent crosslinking between them [17].

Biophysical and computational approaches have shown that RB has a binding preference for positive regions of CLPs both to fibers in solution and hydrogel matrices [17,19]. For instances, although CLPI does not present a specific binding site for RB, the formation of an initial binary ground-state complex between RB and one CLP fiber was shown to be driven by electrostatic interactions [20,21]. The main electrostatic interactions are established between the phenolate and carboxylate groups of RB with side chains of lysine (Lys) residues present in CLPI [20,21]. This initial binary ground-state complex represents a crucial step for the effective covalent crosslinking process of CLPI in the presence of RB. Additionally, RB can establish stabilizing hydrogen bond interactions with the OH side chains present in hydroxyproline (Hyp) residues, which are in the central region of CLPI. These hydrogen bond interactions would help for the positioning and stabilization of RB in the vicinity of the Lys residues. On the other hand, collagen-like peptide II (CLPII) has also been successfully used in PTB therapies in the presence of RB. McTiernan et al. showed that RB forms a stable binary ground-state complex with CLPII and that the RB-CLPII ground-state complex presents a higher stability than the RB-CLPI ground-state complex [21]. The higher stability of the RB-CLPII complex can be because—in addition to the electrostatic interactions established between the phenolate and carboxylate groups of RB and the Arginine (Arg) residue in the specific integrin-binding motif ([GFOGER] domain) of CLPII—RB would also establish another type of interaction with RB [21]. The [GFOGER] domain was proposed as a key factor in integrin binding during the tissue regeneration process. Although CLPII does not present Lys residues in its sequence, the presence of an arginine residue in the [GFOGER] domain represents an RB target to develop new biomaterials that can generate covalent crosslinking. Recently, Wertheimer et al. showed that RB photoactivation can generate efficient arginine-mediated corneal crosslinking [22]. The above experimental information reaffirms the results obtained by molecular dynamics simulations published by Alarcon et al., in which arginine and lysine were indicated as potential electron donors to the RB triplet during PTB [20]. As mentioned above, RB preferentially binds to CLPI and CLPII by electrostatic interactions and forms a binary ground-state complex as the structural basis for the efficient crosslinking of CLP fibers. However, these works do not address the study of the three-dimensional organization of CLPs and which amino acids are involved in stabilizing a ternary complex formed by two CLP fibers and one RB molecule.

In general, the covalent bonds formation process between CLP fibers mediated by RB begins with a structural stabilization of the photosensitizer, mainly motivated by electrostatic interactions. In this scenario, we can separate the covalent bond formation process into two stages: (1) the formation of a structurally and electrostatically favorable ground-state complex, which occurs in the range of tens to hundreds of nanoseconds, validating the use of classical molecular dynamics approach; (2) the electron transfer between RB and the crosslinkable amino acid side chains of CLP fibers; and (3) a stage of covalent bond formation between amino acid side chain radicals. The two last stages must be carried out using quantum methods, starting from a statistically reliable structural conformation. In the present work, by molecular dynamics simulations, we addressed the study of the structural and atomic bases of the micro-organization of CLP-based hydrogels in the presence of RB, and determine the most probable structural configurations between two CLP fibers and one RB molecule.

In addition, by quantum mechanics/molecular mechanics (QM/MM) approaches, our study focused on establishing the framework to treat crosslinking process of CLP. In particular, we focused on quantumly describing the association of RB with CLP fibers before electron transfer occurred. To our knowledge, this crucial crosslinking step has never been described before in detail.

All results together allowed us to establish the energetic and electronic landscape of the interaction of RB with different types of CLP fibers. The present study develops mechanistic knowledge about stabilizing CLP tertiary structures mediated by photosensitive dyes, significantly contributing to the future design of new collagen-like peptide structures for biomedical purposes 

## 2. Materials and Methods

### 2.1. Built System Model 

CLPI, as well as CLPII peptide structures, were previously described by Alarcon et al. [20]. To evaluate a representative hydrogel conformation, we tested a simulation box containing 6 collagen fibrils (3 CLP fibers) with different water boxes for each peptide type. The RB concentration was estimated according to past reports also described by Alarcon [20]. Then, CLPI and CLPII were solvated using the TIP3P water model [23] in a periodic box 46 × 46 × 220 Å for CLPI and 50 × 50 × 210 Å for CLPII. Finally, NaCl counter ions were added to neutralize the systems. The RB-CLPI and RB-CLPII hydrogel models were built on the software VMD v1.93 [24].

### 2.2. Classical Molecular Dynamics Simulations 

All systems were minimized during 30,000 steps followed by five equilibration simulations, in which initial positional restraints were decreased stepwise by 1 ns each, and the final unrestrained simulation was performed by 200 ns (3 replicas). All MD simulations were performed using NAMD v2.12 [25], applying the all-atom CHARMM36 force field [26]. The Langevin thermostat and barostat were used to maintain a constant temperature and pressure at 300 K and 101.325 kPa (1 atm), respectively [27]. Electrostatic interactions were set to 8–9 Å, applying the particle-mesh Ewald method [28] and hydrogen covalent rigid bond restrictions [28,29]. The equations of motion were integrated in 4 fs (femtoseconds) using the repartition mass method [29]. All distances analysis between CLPs residues and RB were performed using tcl/tk and Python languages. 

### 2.3. QM/MM Simulations

To develop QM/MM simulations, 1:1 equilibrated systems of RB-CLPI and RB-CLPII, previously reported by our group [20], were used as starting structures. Then, through the QwikMD tool [30], the QM and MM regions were defined. In detail, the nearest residue (Lys to CLPI and Arg to CLPII) interacting with RB and the dye were set as QM and the rest of the system (peptide, water, and ions) as MM. For the QM system, a −1 charge and multiplicity of three parameters were used, including the radical state of RB. The protocol of QM/MM simulations was (a) 100 steps of minimization, (b) 0.4 ps steps of QM/MM annealing from 0 K to 300 K, and (c) 0.1 ps to QM/MM equilibration. These first three stages were performed using a backbone restraint, as established by QwikMD. Then, we performed QM/MM simulation for 12ps, under an NPT ensemble and with no restraint applied. All simulations were performed at 1 atm of pressure and at 300 K of temperature and a timestep of 0.5 fs. Periodic boundary conditions, Langevin piston barostat and thermostat under standard conditions, and the particle-mesh Ewald method electrostatics were implemented [27,28]. The protein, ions, and water parameters were retrieved from the CHARMM36 [26] force field, and solvation was carried out with the TIP3P water model. We used the B3LYP method and 3–21G basis for electronic description of the quantum mechanics region. All simulations were performed using NAMD 2.14 [31] and Orca software [32,33].

## 3. Results and Discussion

In recent years, the tissue regenerative capacity of CLPI and CLPII in combination with the photoactive dye RB has been proposed. Firstly, RB must bind to CLP fibers forming a ground-state complex. Here, once the dye is activated with green light, the specific interaction of RB with CLP fibers promotes the amino acid side chain radical’s formation. In the present study, we focused on (1) the ability of RB to specifically interact with CLPI and CLPII fibers, (2) determining if RB could bridge and mediate the stabilization of two CLP fibers, and (3) evaluating for the first time the specific association of RB to CLP fibers before electron transfer process occurs through the use of quantic methodologies. For this purpose, the RB-CLPI and RB-CLPII systems were built. Atomistic models of CLPI and CLPII were constructed, taking the structure of collagen type I as a template.

**Figure 1:**
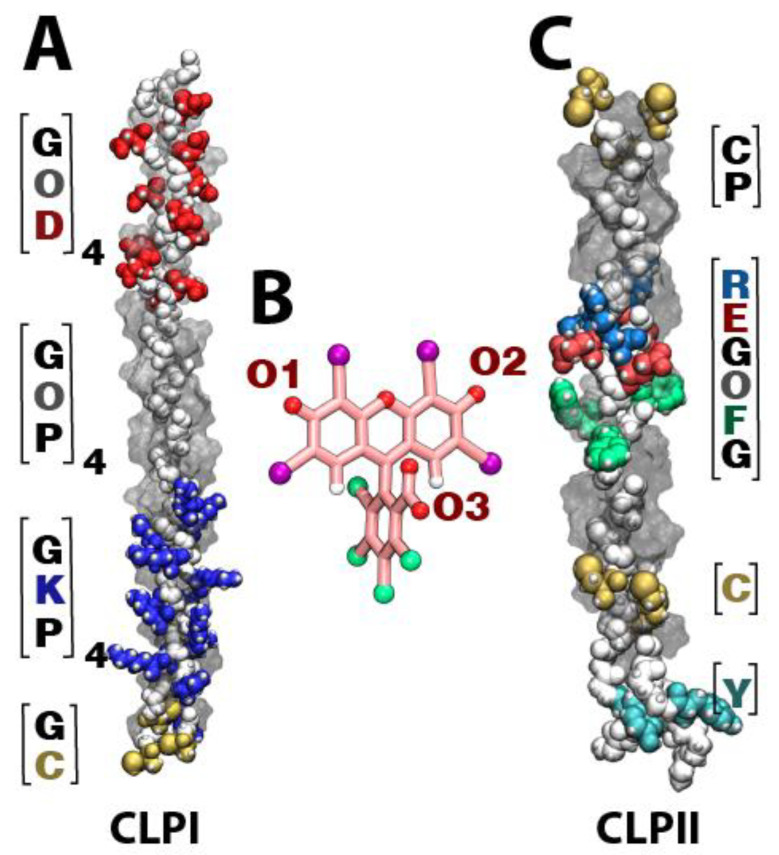
RB structure and structural domains on CLPI and CLPII. (**A**) Three-dimensional structure of CLPI, highlighting negative amino acids in red ([DOG domain]), positively charged amino acids in blue ([PKG] domain) and cysteines in yellow. (**B**) Molecular three-dimensional model of RB. The RB backbone was colored pink; atoms of oxygen, nitrogen, chlorine, and iodide were colored red, blue, green, and purple, respectively. (**C**) Three-dimensional structure of CLPII, highlighting the [GFOGER] domain and the cysteine and tyrosine resides.

Figure 1 shows the different domains that comprise both CLPI (Figure 1A) and CLPII (Figure 1C), as well as the different RB oxygen atoms capable of interacting with collagen fibers (Figure 1B). In the case of CLPI, the fibers can be subdivided into three sections: a negative section ([DOG] domain), neutral section ([POG] domain), and positive section ([PKG] domain). Regarding CLPII, we can highlight a positive central region corresponding to an integrin-binding motif ([GFOGER] domain), and Tyr and Cys residues at its ends (Figure 1C). Regarding RB, the atoms indicated as responsible for interacting with CLP fibers are the oxygen atoms present in the carbonyl groups of the xanthene motif and the carboxylate group present in the aromatic ring (see Figure 1B).

### 3.1. PKG Domain and GFOGER Domain Are Crucial during De Interaction between RB and CLP Fibers

**Figure 2:**
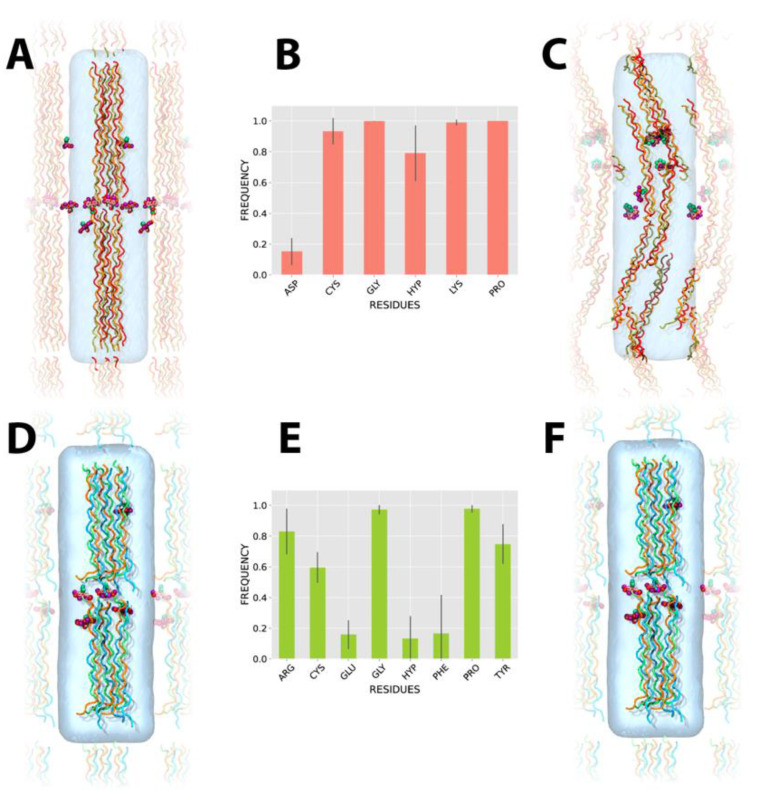
Three-dimensional organization of CLPI and CLPII driven by RB. (**A**,**D**) Initial structural configuration of a CLPI and CLPII system and RB, respectively. (**B**,**E**) Frequency of contact of RB with CLPI and CLPII, respectively. (**C**,**F**) Representative snapshot of the most probable final structural conformation of CLPI and CLPII systems stabilized by the binding of RB.

The CLP systems were built by categorizing three CLP fibers by group. As is shown in Figure 2A,D, CLP, the fibers were arranged in a horizontal position into a box of water, considering a distance that would allow each group not to contact another (Figure 2A for CLPI and Figure 2D for CLPII, respectively). The initial organization was randomly defined, only considering an arrangement such that the final system (considering water) was as small as possible in terms of computational efficiency (110,000 explicit atoms). Ten RB molecules were added to each CLP system, to ensure a high number of contacts between RB and CLP fibers. After minimizing and relaxing the systems under study, 200 ns of production were run, under a three replicas scheme, totalizing 600 ns per each system.

Figure 2C,F represent the final states of the RB-CLPI and RB-CLPII systems, respectively. In terms of the three-dimensional organization of the CLPI fibers, Figure 2C shows that with the simulation production time used, the CLPI fibers can mix and form direct vertical (head-to-tail) interactions between them, which are mediated by electrostatic interactions established between the negative [DOG] domain of one fiber and the positive [PKG] domain of other fibers. Regarding horizontal interactions, molecular dynamics simulations did not show direct interactions between CLPI fibers as neither CLPII but both peptides were stabilized by RB. In both systems, RB mainly established electrostatic interactions with Lys residues in the [PKG] domain of CLPI and with Arg residues in the [GFOGER] domain of CLPII (Figure 2B).

In addition to the electrostatic interactions established by RB with CLPI, the Pro and Gly residues flanking the Lys residue in the [PKG] domain would also play a crucial role in stabilizing the CLPI fiber network by forming stable hydrogen bonds with RB (Figure 2E). Further, we also attribute a high participation in the CLPI-RB stabilization complex to the central Hyp residues present in the central [POG] domain (far from the Asp residues) (Figure 2E). Concordantly, we see that RB has a lower interaction frequency in the vicinity of the [DOG] domain attributable to the high presence of negative Asp residues in this domain. In this case, the electrostatic repulsion between RB and Asp affects any type of interaction between RB and the Gly and Hyp residues of the C-terminal region, emphasizing the importance of Hyp residues in the central region of the peptide. 

In the case of stabilizing the RB-CLPII system, the types of interactions established are also diverse, including prevailing electrostatic interactions between RB and Arg residues. Although the electrostatic interaction between RB and Arg residues present in the [GFOGER] domain is predominant in this system, it is also possible to see that RB establishes pi-stacking interactions with Tyr residues near the C-terminal region of CLPII (Figure 2E). In addition, RB also establishes hydrogen bond interactions with both the central cysteine and the cysteine proximal to the N-terminal region of CLPII. As in the RB-CLPI system, the high frequency of the RB interaction with Gly and Pro residues is because these amino acids in the CLPII sequence are flanking Arg, Tyr, and Cys residues (Figure 2E). Importantly, as in our previous molecular dynamics studies for interactions between CLPI/CLPII single fibers and RB [20], the present work shows that, in a system composed of a larger number of CLP fibers, the preferential interactions of RB remain the same as in single CLP systems.

### 3.2. RB Bridges CLP Fibers Mainly through Electrostatic Interactions

**Figure 3:**
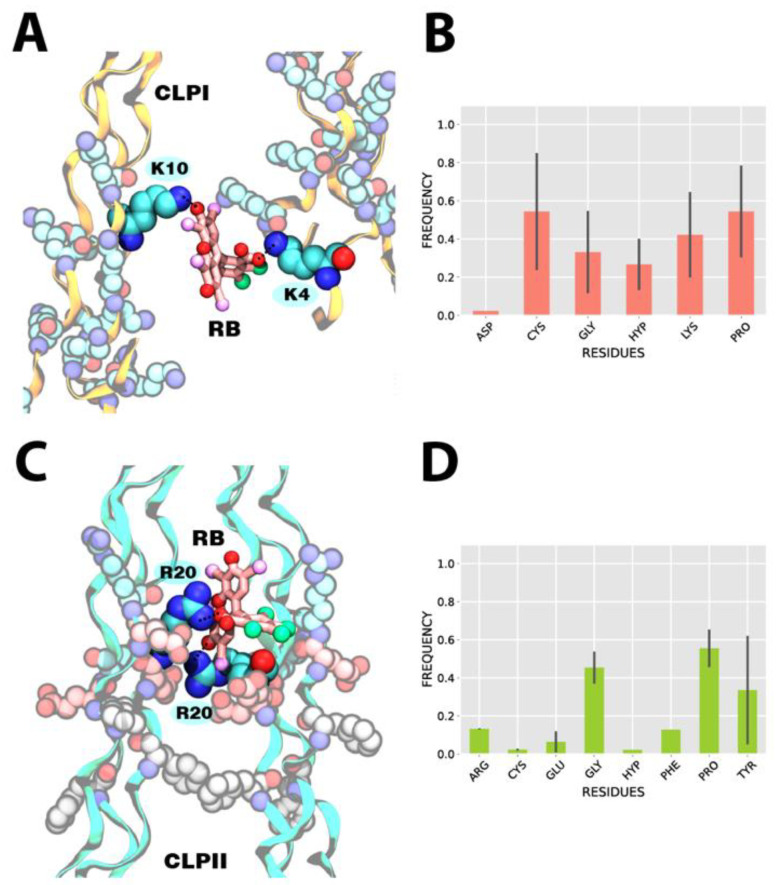
Structural bridge generated by RB with CLPI and CLPII fibers. (**A**) Representative snapshot of a bridge generated by RB between two fibers of CLPI. (**B**) Average contact frequency and standard deviation of residues that allow stabilization of RB-CLPI complex, once a reference interaction with Lys residues has been established. (**C**) Representative snapshot of a bridge generated by RB between two fibers of CLPII. (**D**) Average contact frequency and standard deviation of residues that allow stabilization of RB-CLPII complex once a reference interaction with Arg residues has been established.

Subsequently, our work focused on studying the ability of RB to form inter-molecular bridges between CLPI fibers and CLPII fibers, and thus determine if RB could facilitate the crosslinking process mediating the formation of CLP-RB-CLP ternary complexes (Figure 3).

**Figure 4:**
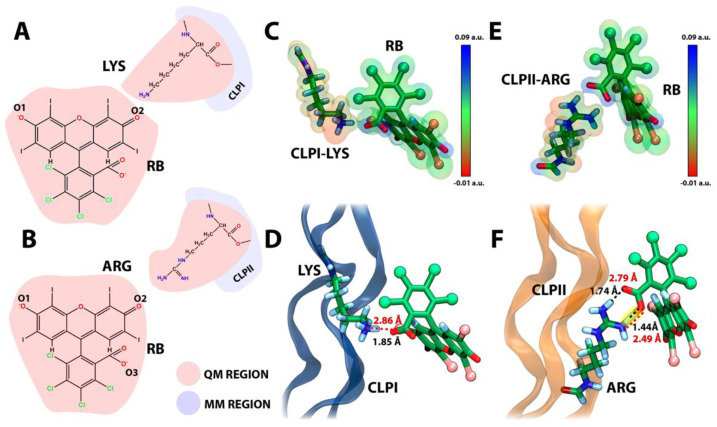
Evaluation of interaction of RB and CLPs with QM/MM method. (**A**,**B**) Representative scheme of region selection for QM/MM calculations in RB-CLPI and RB-CLPII systems, respectively; (**C**) Electronic density of QM region of the last QM/MM simulation frame of RB-CLPI system; (**D**) Structural representation of the last simulation frame for the CLPI-RB system; (**E**) Electronic density of QM region of the last QM/MM simulation frame of RB-CLPII system; and (**F**) Structural representation of the last simulation frame for the RB-CLPII system, yellow highlight emphasizes the formation of a strong hydrogen bond.

In particular, taking as reference the Lys residues in CLPI and Arg in CLPII, and their interaction with the oxygen O1 or oxygen O2 of RB (see Figure 1B and Figure 4A), we analyzed the intermediary role of RB during the crosslinking process of two CLP fibers. Initially, a filtering of the conformations was performed from the last frames of the molecular dynamics simulations of both the RB-CLPI and RB-CLPII systems (last 100 ns). The selected conformations had to meet the following requirements: (1) On the one hand, RB must interact, either through oxygen O1 or through oxygen O2, with a Lys residue in the case of CLPI system, and with a Arg residue in the case of CLPII system. On the other hand, (2) the ground-state complex formed by RB and the first CLP fiber had to have the presence of a second CLP fiber at a distance of no more than 20–25 Å. In this way, the frequency of interaction of oxygen O3 (oxygen carboxylate) of RB with the amino acid residues of the second fiber of CLP was analyzed. Figure 3A shows the most representative structural conformation of the RB-CLPI system, where the RB dye bridges two CLPI fibers via interactions with Lys residues present on both CLPI fibers. Specifically, the ternary ground-state complex CLPI-RB-CLPI is established between the amine group of the Lys 10 of one CLPI fiber and the oxygen O1 of the RB xanthene group (RB:O1-CLPI:K10) while a coupling to the second fiber of CLPI to RB is established between the amine group of Lys 4 and the oxygen O3 (see Figure 1B and Figure 4A) of the carboxylate group of RB (RB:O3-CLPI:K4). In a global analysis, Figure 3B shows the average and standard deviation of the frequency of interaction of each amino acid residue of the second CLPI fiber with the oxygen O3 of RB when RB is attached to the amine group of Lys 10 of the first CLPI. RB establishes an intermolecular bridge in the ground-state ternary complex CLPI-RB-CLPI, mainly by interactions of oxygen O3 of RB with the Lys, Cys, and Pro residues of the second fiber of CLPI. While Lys residues contribute to stabilization through electrostatic interactions, Cys and Pro establish hydrogen bond interactions with CLPI through their backbone.

The same conformational and statistical analysis of the frequency of interactions was carried out for the RB-CLPII system. For the RB-CLPII system, the Arg residues present in the [GFORGER] domain were taken as a reference for the coupling of RB through its oxygen O1 or oxygen O2 (see Figure 1B and Figure 4A) atoms with a first CLPII fiber. Figure 3C shows the most recurrent structural conformation in the RB-CLPII system. RB establishes an intermolecular bridge in the ternary ground-state complex CLPII-RB-CLPII, mainly by interactions of the RB oxygen O2 with the guanidino group of the Arg 20 of a CLPII fiber (RB:O2-CLPII:R20) and by the interaction between the oxygen O3 of the carboxylate group of RB (see Figure 1B and Figure 4A) with the guanidino group of the Arg 20 of a second CLPII fiber (RB:O3-CLPII:R20). In this system, RB preferentially interacts in positive regions of the second fiber of CLPII to initiate the stabilization of the ternary system. Figure 3D shows that the intermolecular bridge mediated by RB is completed due to the participation of residues such as Pro, Gly, and Tyr. While Gly and Pro interact through hydrogen bond interactions of their backbone with the carboxylate group of RB, Tyr promotes the complex’s stabilization through pi-stacking interactions.

Finally, we evaluated the electrostatic VdW and the total binding energy for the most representative ternary complexes’ conformations visited along the MD trajectory. Table 1 shows the five most recurrent conformations in which RB directly interacts with amino acids of different fibers of CLPI. In detail, the nomenclature, i.e., “Lys4-RB-Lys7,” shows that RB is bridging two fibers of CLPI between Lys 4 from one fiber and Lys 7 from the second fiber. Based on that, it is possible to discriminate and categorize five different structural conformations extracted from MD. In detail, for CLPI, the RB reaches the lowest energies when it is bridging to fibers through Lys residues, and the electrostatic energy contributes to the total energy. In the same line, in the RB-CLPII system, the lowest energies are found when RB bridges the CLPII fibers via dye interactions with arginine residues.

### 3.3. RB and CLP’s Interaction in a QM/MM Environment

In recent years, hybrid quantum mechanics/molecular mechanics (QM/MM) has helped us understand the electronic effects in the interactions between proteins and inhibitors [34]. In this sense, the QM/MM method deals with complex systems by treating each part of a given system in different settings, where a buffered region joins the molecular and the quantum mechanics counterparts. Here, we applied the QM/MM approach to address the interaction between the RB and CLPI, as well as CLPII (Figure 4). We used an equilibrated conformation of the complex RB-CLPs extracted from classical molecular dynamics performed in Section 3.2 as a starting point for the QM/MM calculations, prioritizing the electrostatic and conformational stability reached after 200 ns. Therefore, the present study can provide insights into the scaffolding of CLPs structures given by the binding of RB. However, it is important to emphasize that our goal is not to use QM/MM to estimate the total binding affinity but rather to evaluate the likelihood of a strong interaction between the dye and the CLPs.

Figure 4 shows RB’s electrostatic and strong hydrogen bond interaction with CLPI and CLPII obtained from QM/MM simulations. Indeed, Figure 4A,B shows the atoms treated by QM using a B3LYP functional with a 3–21G basis set [35], includes all atoms of RB plus part of the atoms of the Lys 10 and Arg 20 on CLPI and CLPII, respectively. In detail, Figure 4C,E show the initial conformation extracted from the classical molecular simulation discussed in Figure 2 and Figure 3. The electronic density was projected over the VdW volume of both RB and each amino acid, showing correspondence on the opposite charges, which support the initial electrostatic interaction and the stability gained through classical molecular dynamics. Additionally, Figure 4D shows that, in the simulation time considered, RB generates an electrostatic interaction with Lys10 of CLPI, showing a donor–acceptor distance of ~2.8 Å. It can be considered as a moderate-force hydrogen bond and, by consequence, represents a low-barrier electron transfer pseudo-state. This intermediate state could be an antechamber conformational structure to drive the subsequent electron transfer process between the Lys residue and RB to generate the amino acid side chain radicals, producing a crosslinking between the amino acid radicals. On the other hand, Figure 4F shows that, after 12 ps of QM/MM simulation, RB generates a strong hydrogen bond (2.49 Å of the distance between donor–acceptor atoms) through the oxygen of the carbonyl group (RB oxygen 2) to one of the amine groups of the guanidine head of Arg 20 in CLPII. This kind of hydrogen bond was previously reported by Jeffrey et al. [36] and was defined as strong and mostly covalent, where its distance is around 2.2–2.5 Å and has an energy between 14–40 kcal/mol. In the same way as Lys in CLPI, a strong hydrogen bond is established between a positive amino acid side chain (Arg 20) and RB in CLPII. This interaction also decreases the barrier of the electron transfer process, facilitating the radical migration between RB and Arg 20 in CLPII, favoring the subsequent formation of an amino-acid-charged radical specie, which drives the CLP fibers´ crosslinking. Moreover, this interaction was proposed as the initial proton transfer step [37] or electron transfer processes [38], which gives preliminary guidelines about the mechanism involved in RB-mediated CLP crosslinking process for future biomedical studies.

## 4. Conclusions

In summary, our cumulative data point toward an active role of Rose Bengal (RB) in the structural stabilization of hydrogels formed by collagen-like peptides (CLP). By using computational methodologies such as molecular dynamics, we found that the interaction of RB and CLP structures was principally through electrostatics interactions, which in the range of hundreds of nanoseconds, could be efficiently reached and characterized. Indeed, RB generates hydrogen bonds and salt bridges interactions with positive amino acids such as Lys (CLPI) and Arg (CLPII). Additionally, along with the presence of multiple CLP structures, and only with electrostatic interactions to positive amino acids, RB seems to “bridge” and stabilize the structural micro-organization of at least two fibers, triggering the formation of a ternary ground-state complex between CLP fibers. Finally, through using hybrid quantum mechanics/molecular mechanics (QM/MM), it was possible to explore the quantum association process between RB and Lys 10 in an RB-CLPI system, and RB and Arg 20 in an RB-CLPII system. To date, this computational approach in the field of CLP-based biomaterials has not been explored. It is important to emphasize that a detailed QM free-energy determination of the electron transfer process between RB and amino acid side chains or the covalent bond formations between amino acid side chain radicals is needed to thoroughly understand the RB-mediated CLP crosslinking process. The information extracted from this study allows us to delve into the existing knowledge to date on the interaction mechanism of RB with collagen fibers, an essential step prior to the generation of the radicals involved in the crosslinking collagen fibers’ process. In addition to the fact that RB can generate interactions with natural collagen fibers, the use of RB as an initiator of the crosslinking process has also been shown to increase the mechanical stability of commercially available collagen sheets [39]. This study shows for the first time that the formation of strong hydrogen bonds in RB-CLP complexes, in addition to electrostatic interactions, are crucial interactions for the stability of the RB-CLP complex. The present information is relevant from a structural point of view because it will allow for the redesigning of both new CLPs and improved structural and photochemical analogs of RB to enhance the association between these biomedical tools, thus increasing the systems’ stability to achieve a better performance in both in vitro and in vivo models. 

Our findings contribute to the urgent need for a better understanding of the structural components of the hydrogel’s composition, which is undoubtedly needed for the design of safe translational technologies and the development of novel regenerative biomimetic materials.

## Figures and Tables

**Table 1:** Energy evaluation of CLP-RB-CLP ternary complexes interaction formed during molecular dynamics simulations.

CLP	Contact	E. Elect (kcal/molÅ)	VdW (kcal/molÅ)	Total (kcal/molÅ)
CLPI	Lys4-RB-Lys7	−197.541	+2.390	−195.151
	Lys10-RB-Lys4	−192.431	+2.023	−190.408
	Lys10-RB-Pro3	−135.587	−3.991	−139.579
	Lys13-RB-Lys4	−120.513	−4.286	−124.799
	Gly4-RB-Lys4	−53.361	−1.188	−54.599
CLPII	Arg20-RB-Arg20	−164.922	−3.182	−168.104
	Tyr3-RB-Gly1	−103.724	−0.123	−103.846
	Arg20-RB-Glu19	−98.603	−0.824	−99.428
	Arg20-RB-Gly21	−96.080	−0.895	−96.975
	Arg20-RB-Pro23	−25.731	−9.300	−35.031

The energies were calculated using the Namd Energy plugin of VMD [24].
